# ThSCSP_12: Novel Effector in *Tilletia horrida* That Induces Cell Death and Defense Responses in Non-Host Plants

**DOI:** 10.3390/ijms232314752

**Published:** 2022-11-25

**Authors:** Xinyue Shu, Desuo Yin, Juan Liang, Deze Xu, Yuqi Jiang, Ting Xiang, Yuxuan Wang, Chunhai Jiao, Ping Li, Aiping Zheng, Aijun Wang

**Affiliations:** 1 College of Agronomy, Sichuan Agricultural University, Chengdu 611130, China; 2 Food Crop Research Institute, Hubei Academy of Agriculture Sciences, Wuhan 430064, China; 3 Rice Research Institute, Sichuan Agricultural University, Chengdu 611130, China

**Keywords:** rice, *Tilletia horrida*, effector, defense response, interactions

## Abstract

The basidiomycete fungus *Tilletia horrida* causes rice kernel smut (RKS), a crucial disease afflicting hybrid-rice-growing areas worldwide, which results in significant economic losses. However, few studies have investigated the pathogenic mechanisms and functions of effectors in *T. horrida*. In this study, we found that the candidate effector ThSCSP_12 caused cell necrosis in the leaves of *Nicotiana benthamiana*. The predicted signal peptide (SP) of this protein has a secreting function, which is required for ThSCSP_12 to induce cell death. The 1- 189 amino acid (aa) sequences of ThSCSP_12 are sufficient to confer it the ability to trigger cell death in *N. benthamiana*. The expression of ThSCSP_12 was induced and up-regulated during *T. horrida* infection. In addition, we also found that ThSCSP_12 localized in both the cytoplasm and nucleus of plant cells and that nuclear localization of this protein is required to induce cell death. Furthermore, the ability of ThSCSP_12 to trigger cell death in *N. benthamiana* depends on the (RAR1) protein required for Mla12 resistance but not on the suppressor of the G2 allele of Skp1 (SGT1), heat shock protein 90 (HSP90), or somatic embryogenesis receptor-like kinase (SERK3). Crucially, however, ThSCSP_12 induced a defense response in *N. benthamiana* leaves; yet, the expression of multiple defense-related genes was suppressed in response to heterologous expression in host plants. To sum up, these results strongly suggest that ThSCSP_12 operates as an effector in *T. horrida*–host interactions.

## 1. Introduction

As a biotrophic fungal pathogen, *Tilletia horrida* causes rice kernel smut (RKS), a recognized disease problem leading to economic losses because of its harm to hybrid rice crops’ health and quality all over the world [1,2]. The yield loss of hybrid rice crops caused by this pathogen can reach as high as 5–20% annually under suitable conditions [3]. A member of the basidiomycota Tilletiaceae family [4], *T. horrida* mainly infects the floral organs of rice male sterile lines at the flowering stage, producing numerous powdery dark teliospore balls in the kernels during the late phase of infection, which impairs the rice grain-filling process. The teliospore possesses pronounced persistence features, a major factor promoting *T. horrida* inoculation; yet, little is known about the pathogenic mechanisms of *T. horrida* [2].

The effectors of phytopathogens have important roles in promoting infection and suppressing host plant defenses [5,6,7]. Despite this, research on the effector proteins of pathogens is generally scarce, especially in *T. horrida*, for which only two secretory proteins (smut_5844 and smut_2965) capable of triggering non-host cell death and an immune response have been identified as effectors [8]. From the *Ustilaginoidea virens*, responsible for rice false smut, 13 effectors that caused cell death phenotypes in leaves of *Nicotiana benthamiana* were identified [9]. The effector SCRE1 suppresses cell death in *N. benthamiana* induced by the mammalian protein BAX and plays a key role in establishing full virulence of *U. virens* in rice [10]. For smut fungi, such as *Ustilago maydis,* which causes maize common smut, several effectors have been detected: Pit2, See1, Pep1, Cmu1, and Tin2 [11,12,13,14,15].

The mutual recognition between receptor proteins and effectors could suppress plant defenses and thereby facilitate pathogen infection. For example, the RxLR effector RxLR50253 in *Plasmopara viticola* interacts with VpBPA1, a binding partner of ACD11-1, to decrease H_2_O_2_ accumulation, promoting that pathogen’s infection [16]. The effector UvPr1a from *U. virens* suppresses rice immunity by interacting with the suppressor of G2 allele of skp1 (OsSGT1), a positive regulator of innate immunity protein against multiple rice pathogens [17]. Furthermore, the interactions between receptor proteins and effectors are also involved in the activation of plant immune responses [18]. The coiled-coil, nucleotide-binding, and leucine-rich-repeat (CC-NBS-LRR) resistant protein Sr35 in *Triticum monococcum* directly recognizes the effector AvrSr35 from *Puccinia graminis* to form a Sr35 resistosome with the aid of ATP, resulting in the induction of a resistance response [18].

Overall, 597 secreted proteins have been annotated in the *T. horrida* genome’s sequences, some of which are predicted to be effectors [4]. In this study, we show that the putative effector smut_3074 (named ThSCSP_12) of *T. horrida* triggers cell death in *N. benthamiana*, based on a transient expression assay. The predicted secretion signal peptide (SP) and nuclear localization of ThSCSP_12 are both necessary for its ability to induce cell death. Furthermore, the 1-189 amino acid (aa) sequences of ThSCSP_12 are required to trigger cell death in *N. benthamiana*, and ThSCSP_12 also triggers defense responses in *N. benthamiana*. Interestingly, ThSCSP_12 suppresses the expression of multiple defense-related genes and pathways in the host plant. Taken together, these findings enhance our understanding of the molecular mechanisms behind host–*T. horrida* interactions.

## 2. Results

### 2.1. ThSCSP_12 from T. horrida Induces Cell Death in N. benthamiana

**Figure 1:**
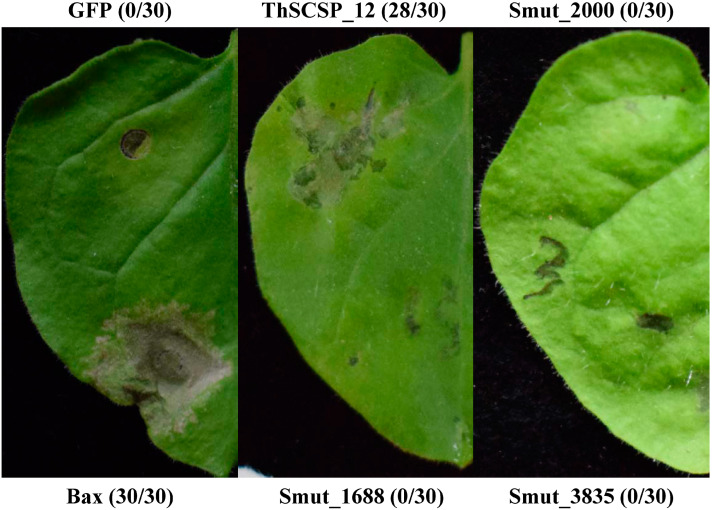
Putative effectors in *Tilletia horrida* induce cell death in *Nicotiana benthamiana* leaves. ThSCSP_12 induced cell death in *N. benthamiana*, whereas three other proteins did not. Green fluorescent protein (GFP) served as a negative control. BAX was the positive control. Numbers e.g., 30/30, indicate that 30 of 30 infiltrated leaves exhibiting cell-death or mottling phenotypes. Representative photos were taken at 4 days post-inoculation (dpi).

Among the 23.2 Mb draft genome sequences of *T. horrida*, 597 potential secreted proteins were annotated [4]. To detect the functions of *T. horrida* effectors, which are small secreted proteins (<400 aa), four effectors that had an N-terminal SP but lacked a transmembrane domain were predicted on http://effectorp.csiro.au/(accessed on 20 April 2022) [19] and successfully cloned, and then each transiently expressed in *N. benthamiana*, to investigate their respective ability to induce cell death. These results showed that ThSCSP_12 (gene ID smut_3074) triggered cell death in *N. benthamiana* leaves at 4 days post-inoculation (dpi) (Figure 1). The green fluorescent protein (GFP) served as a negative control and was used to detect cell death activity by infiltrating *Agrobacterium tumefaciens* into *N. benthamiana* leaves (Figure 1).

### 2.2. ThSCSP_12 Is Conserved in Tilletia Fungi

**Figure 2:**
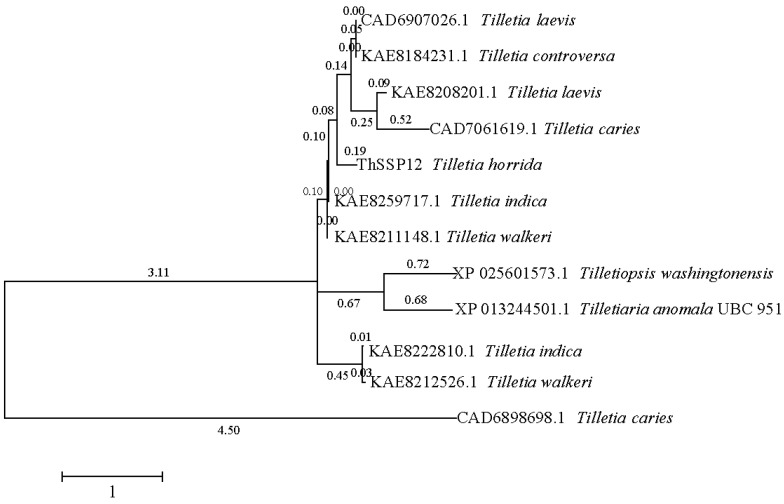
Constructed phylogeny tree of ThSCSP_12 and its homologous secreted proteins.

ThSCSP_12 encodes a 256 aa protein, which contains a predicted N-terminal SP (19 aa) and eight cysteine residues; however, a conserved domain was not found in its coding region. It has been reported that effector proteins are conserved in plant pathogen fungi [20]. We also found several homolog genes of ThSCSP_12 in *Tilletia* fungi, such as *T. indica*, *T. walkeri*, *T. caries*, *T. controversa*, and *T. laevis* (Figure 2). Interestingly, the homolog genes of ThSCSP_12 were not detected in other fungi that are phytopathogens. This result implied that the potentially secreted protein ThSCSP_12 is conserved exclusively in *Tilletia* fungi, which are important causal agents of disease in crops. Hence, we speculated that ThSCSP_12 plays a special role in rice–*T. horrida* interactions.

### 2.3. Functional Analyses of the SP Predicted in ThSCSP_12

**Figure 3:**
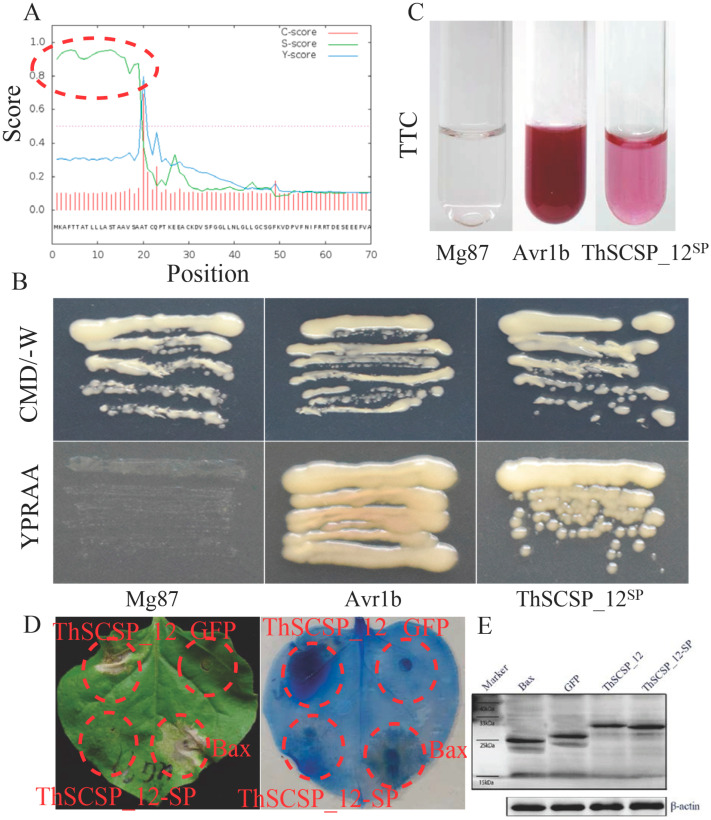
The signal peptide (SP) of ThSCSP_12 is functional. (**A**) The SP sequence of ThSCSP_12. (**B**) Functional validation of the SP of ThSCSP_12 using the yeast invertase secretion assay. All transformed YTK12 yeast strains were grown on YPRAA media with raffinose as the sole carbon source. N-terminal sequences of *Phytophthora sojae* Avr1b and *Magnaporthe oryzae* Mg87 were used as positive and negative controls, respectively. The untransformed YTK12 did not grow on either CMD-W or YPRAA medium. Yeast growth on CMD-W media was equally viable among the transformed strains. Mg87: negative control Mg87 SPs; Avr1bSP: positive control Avr1b SPs; ThSCSP_12^SP^: SPs of ThSCSP_12. (**C**) Enzymatic activity of invertase was detected by the reduction of 2, 3, 5-triphenyltetrazolium chloride (TTC) to insoluble red-coloured 1, 3, 5-triphenylformazan (TPF). The negative control YTK12 strain carrying the ThSCSP_12’s SP did not degrade TTC to red-colored TPF. By contrast, the positive control YTK12 strain carrying Avr1b’s SP, as well as the YTK12 strain carrying Avr1b’s SP, could degrade TTC to red-colored TPF. (**D**) The predicted SP of ThSCSP_12 is required to induce cell death. (**E**) Expressed proteins were detected in the leaves of *Nicotiana benthamiana* following the western blot experiments. ThSCSP_12^SP^ denotes the SP sequence of ThSCSP_12; ThSCSP_12-SP denotes the sequence of ThSCSP_12 without the SP.

We used a yeast secretion assay to analyze the secretory function of the signal peptide (SP) predicted in ThSCSP_12 [20]. The ThSCSP_12^SP^ (SP nucleotide sequence of ThSCSP_12; Figure 3A) was fused in frame with the truncated pSUC2 gene, which encodes invertase without the SP. This result revealed that the fusion protein with ThSCSP_12^SP^ and the positive control (i.e., fusion protein with the secretion signal of *Phytophthora sojae* Avr1b) was secreted from the transformed yeast YTK12 (Figure 3B), whereas the N-terminus of Mg87 in *M. oryzae* (a negative control) was not secreted from the transformed yeast YTK12 (Figure 3B). The color reaction of 2, 3, 5-triphenyltetrazolium chloride (TTC) was further used to identify the secretory function of ThSCSP_12^SP^; this showed that the secreted invertase was able to reduce the colorless TTC into insoluble red 1, 3, 5-triphenylformazan (TPF) (Figure 3C). Altogether, these results demonstrated that ThSCSP_12^SP^ leads to the secretion of invertase and functionally secreted proteins.

Furthermore, the SPs of many pathogenic fungi effectors are necessary for inducing cell death in plants [21]. Thus, to clarify whether ThSCSP_12^SP^ is a prerequisite for the ability of ThSCSP_12 to induce cell death, we transiently expressed ThSCSP_12 lacking the SP (i.e., ThSCSP_12-SP) in *N. benthamiana* leaves. This result revealed that ThSCSP_12-SP was incapable of inducing cell death (Figure 3D). Western blotting confirmed that ThSCSP_12-SP was expressed in the infiltrated *N. benthamiana* leaves (Figure 3E). These results suggested that the SP is needed for ThSCSP_12 to trigger cell death in *N. benthamiana*.

### 2.4. Nuclear Localization of ThSCSP_12 Is Required to Induce Cell Death

**Figure 4:**
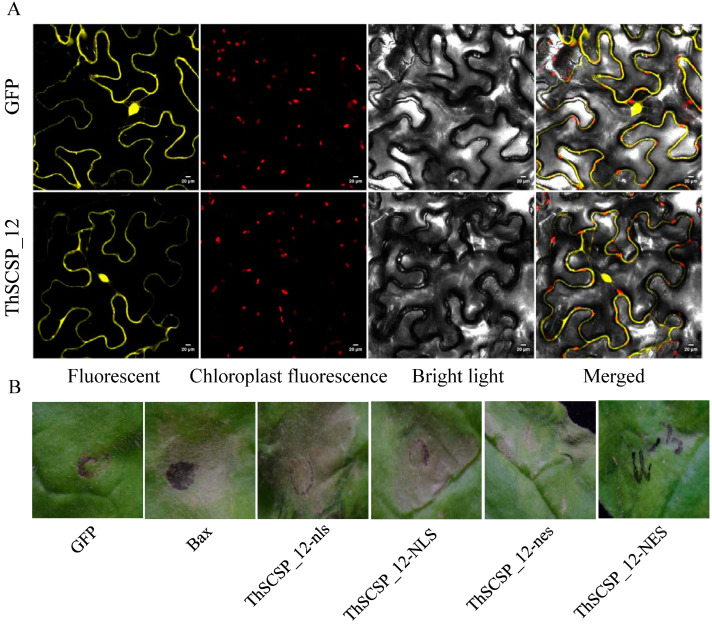
Nuclear localization of ThSCSP_12 is required to induce cell death. (**A**) Subcellular localization of ThSCSP_12-SP transiently expressed in Nicotiana benthamiana leaves. The vector PHB carrying YEP served as a control. Bars = 20 μm. (**B**) Cell death triggered by nuclear export signal (NES) targeted ThSCSP_12 is both delayed and weak. Photographs were taken at 4 days post-inoculation (dpi). Each treatment was performed using at least three biological replicates.

To explore the subcellular localization of ThSCSP_12, we transiently expressed 2, 35S:ThSCSP_12-SP -GFP in tobacco leaf epidermis cells. The transient expression of 2, 35S:-YFP served as the control. The ThSCSP_12-SP-GFP protein localized to the plasma membrane, cytoplasm, and nucleus of the transiently transformed tobacco leaf epidermis cells when compared with the control (Figure 4A). Nuclear localization is known to be important for the ability of some pathogenic fungi effectors to induce cell death in plants [22]. To test whether the nuclear localization of ThSCSP_12 was essential for triggering cell death, we forced ThSCSP_12 to locate to the nucleus or cytoplasm in plant cells by fusing to it a nuclear localization signal (NLS) or a nuclear export signal (NES) and detected their cell-death-inducing ability, respectively. ThSCSP_12 attached to non-functional NLS (nls) or NES (nes) were included as controls [23]. The transient expression of these sequences demonstrated that NES-ThSCSP_12 led to a pronounced attenuation of cell-death-inducing activity in *N. benthamiana* leaves (Figure 4B). However, NLS- ThSCSP_12 and ThSCSP_12 each resulted in a strong cell death phenotype (Figure 4B). In addition, nes and nls did not alter the action of ThSCSP_12 in inducing cell death (Figure 4B). Collectively, these results suggested that the localization of ThSCSP_12 in plant cell nucleus is critical for its cell-death-inducing ability.

### 2.5. ThSCSP_12-Triggered Cell Death in N. Benthamiana Depends on RAR1 but Not SGT1, HSP90, or SERK3/Bak1

**Figure 5:**
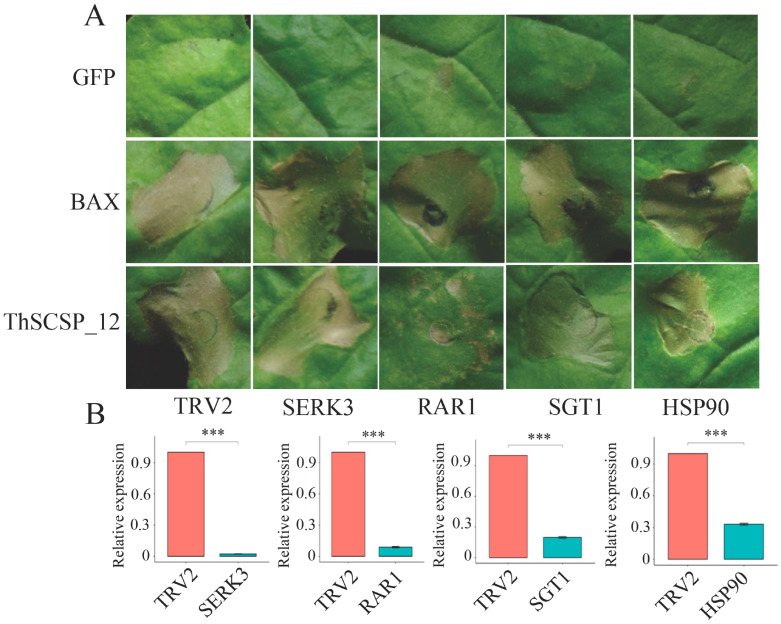
The RAR1 protein is required for cell death induced by ThSCSP_12. (**A**) Representative photographs of ThSCSP_12-induced cell death in silenced *Nicotiana benthamiana* leaves at 4 days post-inoculation (dpi). At 20 dpi, *Agrobacterium tumefaciens* carrying ThSCSP_12 was infiltrated into the upper leaves of silenced plants of tobacco rattle virus (TRV) constructs. The control consisted of un-silenced plants. (**B**) The transcript expression levels of RAR1, SGT1, HSP90, and SERK3 in corresponding silenced plants were analyzed by qRT-PCR. The constitutively expressed gene *NbActin* was used as internal reference. Statistical analysis was performed by one-way ANOVA, followed by Tukey’s multiple comparison test. Error bars are the standard deviation (SD) of three biological replicates (*** *p* < 0.001).

The suppressor of G2 allele of Skp1 (SGT1), heat shock protein 90 (HSP90), and the (RAR1) protein required for Mla12 resistance are three important factors contributing to resistance induced by the R protein [24,25]. Thus, the virus-induced gene silencing (VIGS) assay against the corresponding genes encoding them in *N. benthamiana* was performed to clarify the role of SGT1, HSP90, and RAR1 in cell death triggered by ThSCSP_12, for which Bax served as a positive control. The results show that ThSCSP_12 induced cell death in the SGT1- and HSP90-silenced plants, but the cell death phenotype was not discernible in the RAR1-silenced plants (Figure 5A). The expected low expression of these genes was also confirmed in silenced plants by using quantitative real-time reverse transcription PCR (qRT-PCR) (Figure 5B). Furthermore, we also identified whether somatic embryogenesis receptor-like kinase SERK3/Bak1 could be involved in ThSCSP_12-induced cell death in *N. benthamiana*. SERK3/Bak1 is a key regulator in the pathogen-associated molecular pattern (PAMP) [26]. We found that ThSCSP_12 also induced cell death in SERK3/Bak1-silenced plants (Figure 5A). Therefore, these results revealed that ThSCSP_12-triggered cell death depends on RAR1 but not SGT1, HSP90, or SERK3/Bak1.

### 2.6. The 1-189 Amino Acid Fragment of ThSCSP_12 Is Sufficient for Its Cell-Death-Inducing Activity

**Figure 6:**
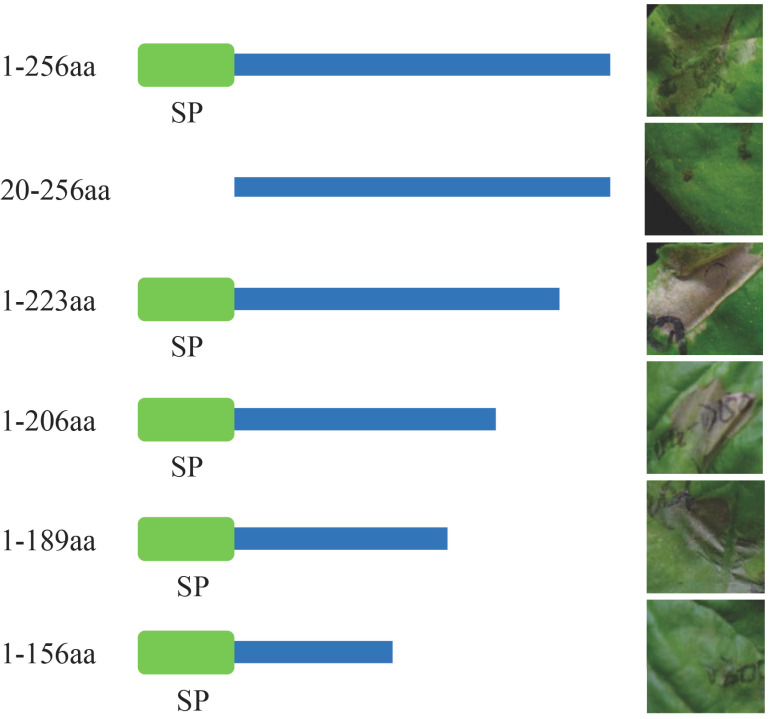
The 1-189 amino acid (aa) fragment of ThSCSP_12 is sufficient to induce cell death in *Nicotiana benthamiana*. Various truncated mutants of ThSCSP_12 were constructed and transiently expressed by agroinfiltration in *N. benthamiana* leaves. Photographs were taken 4 days after agro-infiltration.

To further clarify the functional sequence area of ThSCSP_12 during cell death induction, the deletion mutants of the C-terminal of the ThSCSP_12 protein were generated and tested for their ability to induce cell death via agro-infiltration of *N. benthamiana* leaves. These results showed that truncated 33, 50, and 67 aa sequences still preserved the ability to induce cell death in *N. benthamiana*, whereas truncated 100 aa sequences failed to trigger cell death (Figure 6). Combined with the SP result above, we inferred that the 1 -189 aa sequences are required to confer to ThSCSP_12 its ability to induce cell death (Figure 6).

### 2.7. ThSCSP_12 Expression during T. horrida Infection

**Figure 7:**
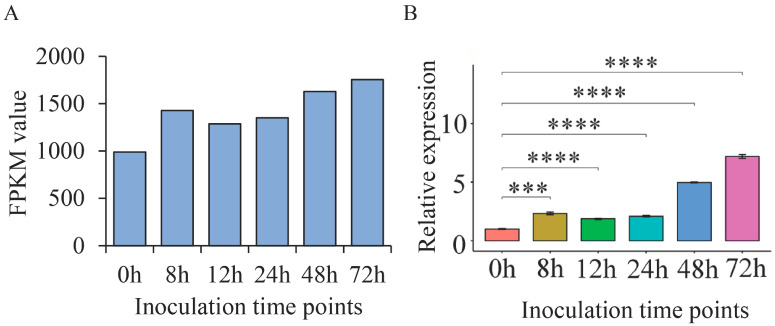
Expression of ThSCSP_12 during *Tilletia horrida* infection of the kernel smut–susceptible rice cultivar 9311A. (**A**) The transcriptome data. (**B**) The qRT-PCR analysis of ThSCSP_12. Rice kernels inoculated with *Tilletia horrida* were collected at 8, 12, 24, 48, and 72 hpi (hours post-inoculation) for gene expression analyses using qRT-PCR. UBQ expression was used as an internal reference for normalizing expression levels within the samples. Statistical analysis was performed by one-way ANOVA, followed by Tukey’s multiple comparison test. Error bars are the standard deviation (SD) of four independent replicates (*** *p* < 0.001; **** *p* < 0.0001).

The expression of effector genes in phytopathogens is typically induced during their infection of host plant tissues [27]. As expected, the expression of ThSCSP_12 was induced, being up-regulated by *T. horrida* infection according to the transcriptome data (Figure 7A) [4]. To verify these data used for that inference, we further tested ThSCSP_12 expression at different inoculation time points (8, 12, 24, 48, and 72 h) in the rice cultivar 9311A, which is susceptible to *T. horrida*, using qRT-PCR. These results showed that ThSCSP_12 was up-regulated in response to inoculation with *T. horrida*, thus consistent with the transcriptome data (Figure 7B). These results demonstrated that ThSCSP_12 is up-regulated at the early stage of *T. horrida* infection and that it has an essential role to play in *T. horrida*–rice interactions.

### 2.8. ThSCSP_12 Triggers Immunity Responses in N. benthamiana

**Figure 8:**
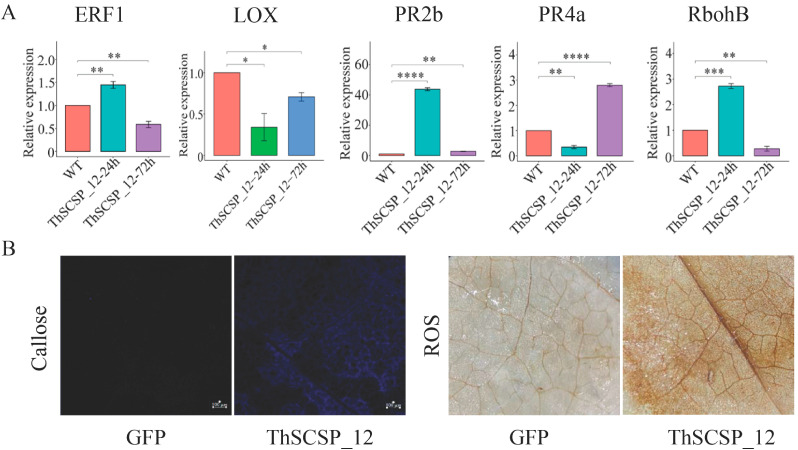
ThSCSP_12 triggers plant immunity responses in *Nicotiana benthamiana*. (**A**) Expression of genes related to plant immunity in *N. benthamiana* leaves transiently expressing ThSCSP_12 at 24 and 72 hpi (hours post-inoculation). Statistical analysis was performed by one-way ANOVA, followed by Tukey’s multiple comparison test. Error bars are the standard deviation (SD) of four independent replicates (* *p* < 0.05; ** *p* < 0.01; *** *p* < 0.001; **** *p* < 0.0001). (**B**) Accumulation of reactive oxygen species (ROS) and deposition of callose in *N. benthamiana*. For the observation of callose; bars = 20 μm. These experiments were replicated three times with six leaves per biological replicate.

To clarify whether ThSCSP_12 activates the immune response in *N. benthamiana*, the expression levels of ethylene response factor 1 (*ERF1*), lipoxygenase (*LOX*), respiratory burst oxidase homolog protein B (*RbohB*), and two pathogenesis-related (PR) protein genes *PR2b* and *PR4a* during ThSCSP_12 infiltration were evaluated via qRT-PCR [28,29,30]. We found that the expression of *ERF1*, *RbohB*, and *PR2b* was up-regulated at 24 h, and that of PR4a was strongly induced by ThSCSP_12 at 72 h (Figure 8A). This indicated that ThSCSP_12 plays a key role in activating early defense responses in non-host plants. Reactive oxygen species (ROS) accumulation and callose deposition are both important early defense responses in plants against pathogen infection [31,32]. Thus, we also detected the ability of ThSCSP_12 to trigger hydrogen peroxide (H_2_O_2_) and callose deposition in *N. benthamiana* leaves. These results showed that H_2_O_2_ was activated by ThSCSP_12 at 4 dpi vis à vis the control, and extensive callose deposition was found at 24 h after inoculation of the *N. benthamiana* leaves (Figure 8B).

### 2.9. Transcriptome Analysis of ThSCSP_12 Transgenic Rice

**Figure 9:**
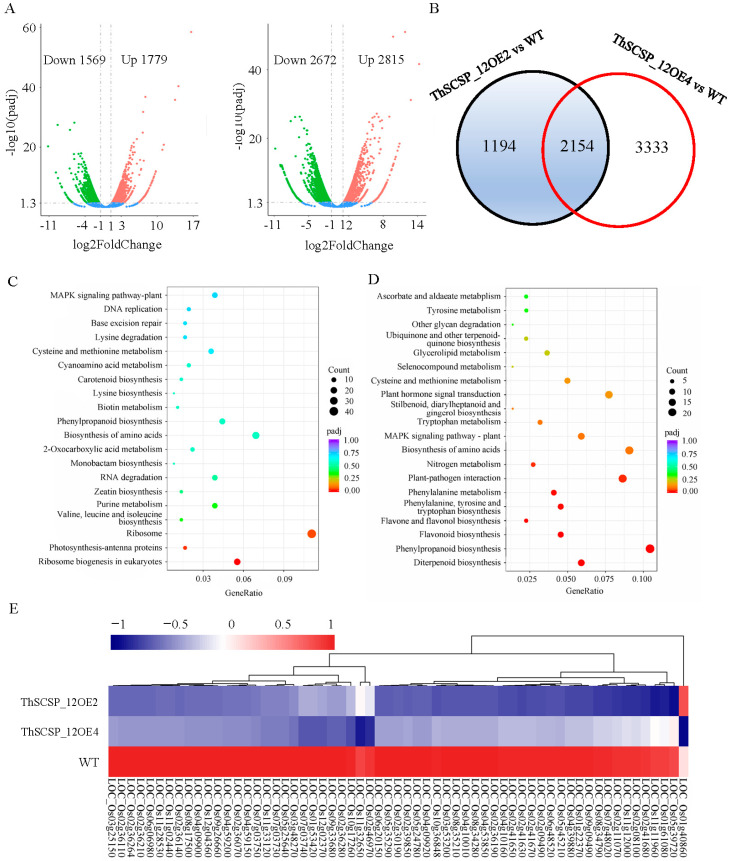
Analysis of RNA-seq data for the ThSCSP_12-OE and wild-type (WT) plants. (**A**) The number of differentially expressed genes (DEGs) in ThSCSP_12-OE2 and ThSCSP_12-OE4 plants compared with WT. (**B**) Venn diagrams showing the overlap of DEGs in ThSCSP_12-OE2 and ThSCSP_12-OE4 plants. (**C**) KEGG enrichment analysis of up-regulated DEGs in both ThSCSP_12-OE2 and ThSCSP_12-OE4 plants. For the dots, their size and color represent the number of DEGs and their adjusted *p*-value, respectively. (**D**) KEGG enrichment analysis of the down-regulated DEGs in both ThSCSP_12-OE2 and ThSCSP_12-OE4 plants. For the dots, their size and color represent the numbers of DEGs and the q-value, respectively. (**E**) Expression of DEGs enriched in the pathways for phenylpropanoid biosynthesis, diterpenoid biosynthesis, flavonoid biosynthesis, phenylalanine metabolism, and plant- pathogen interaction in the ThSCSP_12-OE2 and ThSCSP_12-OE4 plants. Four of those are involved in reactive oxygen metabolism and cell death.

To explore the potential function of ThSCSP_12 in *T. horrida*–rice interactions, two ThSCSP_12 transgenic rice lines, ThSCSP_12OE2 and ThSCSP_12OE4, were obtained (Supplementary Figure S1). We analyzed the changes to gene expression in ThSCSP_12 transgenic rice plants in comparison with the wild type (WT) using transcriptome sequencing technology. The result showed that 3348 (1569 down-regulated, 1779 up-regulated) and 5487 (2672 down-regulated, 2815 up-regulated) differentially expressed genes (DEGs) were identified in ThSCSP_12OE2 and ThSCSP_12OE4 lines when compared with the WT lines, respectively (Supplementary Tables S1 and S2; Figure 9A). Among these DEGs, 2154 were found in both ThSCSP_12 transgenic lines, consisting of 1087 up-regulated and 1067 down-regulated genes (Figure 9B). This result demonstrated that the expression of multiple genes was induced by ThSCSP_12 heterologously expressed in host plants.

To detect the biosynthesis pathways in which these DEGs are involved, a Kyoto Encyclopedia of Genes and Genomes (KEGG) pathway enrichment analysis was carried out. This showed that those 1087 up-regulated/1067 down-regulated DEGs in both ThSCSP_12OE2 and ThSCSP_12OE4 lines were, respectively, enriched in 103/96 pathways (Supplementary Tables S3 and S4). We further analyzed the top 20 enriched pathways, finding that several defense-related pathways, such as phenylpropanoid biosynthesis, diterpenoid biosynthesis, flavonoid biosynthesis, phenylalanine metabolism, and plant–pathogen interaction, were significantly enriched (padj value < 0.05) only in these down-regulated DEGs (Figure 9C,D). In addition, 19, 23, 10, and 9 DEGs were enriched in the plant–pathogen interaction, phenylpropanoid biosynthesis, flavonoid biosynthesis, and phenylalanine metabolism pathways, respectively, whose expression patterns are shown in Figure 9E. Among the 19 DEGs enriched in the plant–pathogen interaction, we distinguished 4 reactive-oxygen-metabolism- and cell-death-related genes, namely OsrbohB, OsrbohE, OsrbohD, and Osrboh8, whose expression was significantly induced and down-regulated by ThSCSP_12 (Figure 9E). We also confirmed the accuracy of the expression patterns of DEGs generated by RNA-seq using RT-qPCR. For this, eight genes involved in plant defense pathways were selected to analyze their expression levels in WT and ThSCSP_12-expressing rice plants (Supplementary Figure S2). The expression levels of these eight genes in the RNA-seq results were consistent with the RT-qPCR results (Supplementary Figure S1), suggesting our study’s transcriptome analysis results are reliable.

## 3. Discussion

Effectors are usually virulence proteins in phytopathogens, being critically involved in the interaction between plants and their pathogens [27,33]. Dozens of effectors have been reported on from various plant pathogens during the last decade through high-throughput sequencing studies [34,35]. Thus, with the complete sequencing of the *T. horrida* genome, many of its genes, which encode small cysteine-rich secreted proteins, were predicted to be effectors [4]. However, the elucidation of these effectors’ functions in *T. horrida*–rice interactions has progressed slowly, and only two effectors, smut_2965 and smut_5844, have been reported to induce cell death and a defense response in *N. benthamiana* [8]. In the present study, we found that ThSCSP_12 may act as an effector, playing a key role in activating the plant defense response. Importantly, those host target genes that interact with ThSCSP_12 were predicted by the RNA sequencing analysis. This helps us understand how ThSCSP_12 participates in rice–*T. horrida* interactions.

The expression of ThSCSP_12 was induced and up-regulated during the early stages of *T. horrida* infection, similar to most effectors of plant pathogenic fungi, such as RsSCR10 and RsIA_NP8 in *R. solani* AG1-IA, which causes rice sheath blight [36,37]. The SP is required for ThSCSP_12 to trigger cell death in *N. benthamiana*. This phenomenon was also found for uan2, a reported effector protein in *T. horrida* [8], as well as for RsSCR10 and RsIA_NP8 [36,37]. In addition, multiple effector proteins from *U. virens*, such as UV_44, UV_1338, UV_4753, and UV5517, were observed to promote cell death in *N. benthamiana,* and this depended on their SP [9]. Accordingly, we believe that these effector proteins might have common characteristics and that they function in the extracellular space [38,39].

All kinds of domains can be found in the effector proteins of plant pathogens, contributing significantly to their pathogenic functions. For example, some RxLR effectors carry RxLR motifs, and some contain repeat motifs that are defined as repeat-containing proteins [5]. The effector protein XopN from *Xanthomonas euvesicatoria* could interact with the positive regulators of host immunity TFT1 by virtue of its HEAT/armadillo-like repeats [40]. Yet, no domain was detected in the coding area of ThSCSP_12, and we found that the 1- 597 nucleotide sequences are required for its ability to induce cell death in *N. benthamiana*. Therefore, we suspect that ThSCSP_12 is a new effector in plant pathogenic fungi, playing a key role in the specialization interaction of rice and *T. horrida*. This clearly merits further study.

The complex consisting of HSP90, SGTI, and RAR1 is known to stabilize and sustain R-protein-mediated ETI responses [25,41]. For an RXLR effector PlAvh142 from *Peronophythora litchi*, its ability to trigger cell death is dependent on HSP90, SGTI, and RAR1 [42]. In the case of *P. litchii* AVR-blb2 or PITG_22798, only SGT1 is required to sustain their cell-death-inducing activity [43,44]. In the present study, the silencing of RAR1 in *N. benthamiana* resulted in the suppression of ThSCSP_12-triggered cell death, indicating that ThSCSP_12-induced cell death is somehow regulated by the RAR1 protein. Together, these results illustrate the fact that plants possess multiple pathways for regulating cell death in response to different effectors.

In addition, ThSCSP_12 suppressed multiple defense-related pathways in its host plant, such as phenylpropanoid biosynthesis, diterpenoid biosynthesis, flavonoid biosynthesis, phenylalanine metabolism, and plant–pathogen interaction pathways, which shows that these biological processes figure prominently in the ecological interaction of *T. horrida* and rice [45,46]. Interestingly, these pathways were activated by *T. horrida* infection in the resistant rice male sterile line 4766A [47]. Furthermore, the transcription levels of four reactive-oxygen-metabolism- and cell-death-related genes OsrbohB, OsrbohE, OsrbohD, and Osrboh8 [48,49] were suppressed by expression of ThSCSP_12 in the host plant. These results suggest that ThSCSP_12 could be part of a network that participates in the response to host defense and ensures fungal colonization during the infection process. We speculate that ThSCSP_12 suppresses the host defense response and promotes *T. horrida* infections, inhibiting the ROS burst. If so, in this way, it would be similar to the effector protein UvCBP1 from *U. virens*. UvCBP1 is able to suppress the rice defense response and promote *U. virens* infection by interacting with the scaffolding protein OsRACK1A, leading to ROS production being distinctly inhibited [50].

## 4. Materials and Methods

### 4.1. Fungal Strains, Plant Materials, and Growth Conditions

The *T. horrida* strain JY-521 was saved at the College of Agronomy of Sichuan Agricultural University. The *A. tumefaciens* GV3101 was grown in YEP medium (NaCl 5 g, 1% tryptone 10 g, yeast extract 10 g, and distilled water 1000 mL). The yeast strain YTK12 was cultivated using YPDA medium (glucose 20 g, peptone 20 g, yeast extract 10 g, agar 20 g, adenine hemisulfate 0.03 g, and distilled water 1000 mL). The antibiotics and their concentrations were as follows: ampicillin 100 μg/mL^–1^, kanamycin 100 μg/mL^–1^, and rifampin 25 μg/mL^–1^. Rice cultivar 9311A and Nipponbare were stored at the College of Agronomy of Sichuan Agricultural University. The tobacco (*N. benthamiana*) plants were grown under a 12 h/12 h night–day photoperiod at 23 °C with 60% relative humidity.

### 4.2. Screening of Effectors

SignalP 4.11 and TMHMM server v2.02 tools were, respectively, used to predict the signal peptides and transmembrane helices of secreted proteins [51,52]. To predict the effectors, the website http://effectorp.csiro.au/ (accessed on 20 April 2022)was used; of the small secreted proteins (<400 aa) predicted to be effectors, those containing an N-terminal SP and lacking a transmembrane domain were considered as candidate effectors [19].

### 4.3. RNA Isolation and Plasmid Construction of T. horrida Candidate Effector Genes

The total RNA of *T. horrida* strain JY-521 was extracted using the Fungal RNA Kit (Omega, Biel, Switzerland), and the cDNA was generated by the Transcriptor First Strand cDNA Synthesis Kit (Roche, Basel, Switzerland). TransStart FastPfu Fly DNA Polymerase (TransGen Biotech, Beijing, China) was used to amplify the full-length nucleotide sequence of each candidate effector protein. Restriction enzymes and ClonExpress enzymes (Vazyme Biotech, Nanjing, China) were used as per the manufacturer’s instructions. CE Design v 1.03 was used to design the primers of these genes, according to the *T. horrida* strain JY-521 genome sequences, and included a BamHI site and a StuI site. The primer sequences used are listed in Supplementary Table S5. The cDNA sequences of candidate effector genes were cloned into the 35S-PMDC32 expression vector.

### 4.4. A. grobacterium–Mediated Transient Expression

The expression vector 35S-PMDC32 harboring a given candidate effector gene was transformed into the *A. tumefaciens* strain GV3101. The bacteria were centrifuged at 5000× *g* for 5 min and then resuspended in MES buffer (200 μM acetosyringone, 10 mM MgCl_2_, and 10 mM MES [pH 5.6]). The OD600 of the bacterial suspensions was adjusted to 0.8 and incubated for 3 h at room temperature in darkness. At the five-leaf stage, we used the *A. tumefaciens* strain possessing the construct vector to infect the leaves of *N. benthamiana*. The 35S-PMDC32 vectors carrying the GFP and Bax genes were used as a negative and positive control, respectively. A total of 30 leaves from different plants were infiltrated for each treatment. The cell death phenotypes were detected at 4 dpi (days post-inoculation).

### 4.5. Function Validation of the SP

The yeast secretion assay and TTC experiment were used to analyze the secretory function of the ThSCSP_12′s SP. The ThSCSP_12^SP^ sequence was amplified with specific primers by PCR (Supplementary Table S5) and connected to a pSUC2 vector. The pSUC2 vector with ThSCSP_12^SP^ was transformed into the yeast strain YTK12 using the Frozen-EZ Yeast Transformation II Kit (Zymo Research, Irvine, CA, USA). Then, the yeast was cultivated on CMD-W (6.7 g yeast N base without amino acids, 0.75 g tryptophan dropout supplement, 20 g sucrose, 1 g glucose, 15 g agar, and 1000 mL distilled water, pH 5.8) and YPRAA solid media (10 g yeast extract, 20 g peptone, 20 g raffinose, 2 μg antimycin A, l5 g agar, and 1000 mL distilled water, pH 5.8). The invertase activity was observed by the reduction of TTC to an insoluble, red-colored 1, 3, 5-triphenylformazan (TPF). The transformants were grown on CMD/-W liquid medium at an OD600 of 0.3 for 10 h. Next, the cell suspension (approximately 1.5 mL) was collected and re-suspended with 250 μL of 10 mM acetic acid–sodium acetate buffer, 500 μL of a 10% sucrose solution (*w*/*v*), and 750 μL of sterile distilled water, at 37 °C for 10 min. After centrifugation at 12,000× *g* for 1 min, 100 μL of the supernatant was transferred into a glass test tube containing 900 μL of a 0.1% TTC solution and placed at room temperature for 5 min.

### 4.6. Protein Extraction and Western Blotting

We collected the *N. benthamiana* leaves at 2 days after infiltration and ground them in liquid nitrogen. The protein was extracted from the leaves by using a single-step plant active protein extraction kit (Sangon Biotech, Shanghai, China) and following the manufacturer’s instructions. The extracted proteins were separated using 10% sodium dodecyl sulfate–polyacrylamide gel electrophoresis gels and then transferred onto nitrocellulose membranes. These membranes were blocked for 1 h at room temperature by 5% milk in TBS-T buffer (50 mM Tris–HCl, pH 7.5; 150 mM NaCl; 0.05% Tween 20) and incubated with the anti-FLAG antibody (1:5000 dilution). Then, all membranes were washed three times with TBS-T buffer. We further used an eECL Western blotting kit (CWBio) to observe the immunoblots and photographed them on X-ray films.

### 4.7. Deletion Mutagenesis of ThSCSP_12

PCR was used to obtain the truncated nucleotide sequences of ThSCSP_12, after which the PCR products were cloned into 35S-PMDC32 vectors. The primer sequences used are listed in Supplementary Table S5.

### 4.8. Subcellular Localization

The ThSCSP_12-SP sequence was connected to the PHB-YFP vector and then transformed into *A. tumefaciens* GV3101. The *N. benthamiana* leaves were infiltrated with transformed *A. tumefaciens* GV3101, as described previously. Subcellular localization was detected by laser confocal fluorescence microscopy 2 days after infiltration.

### 4.9. VIGS Assay in N. benthamiana

The *A. tumefaciens* GV3101 strains with pTRV1 and pTRV2 genes were cultivated on YEP media for 36 h and their biomass collected by centrifugation, then suspended using the infiltration medium mentioned above, and mixed in a 1:1 ratio with an OD600 = 0.6 for each strain. At the four-leaf-stage, the *N. benthamiana* plants were infiltrated using syringes. At 20 days after infiltration, the gene silencing efficiency of NbHSP90, NbSERK3, NbRAR1, and NbSGT1 was determined by RT-qPCR. Four biological replicates were performed. Statistical analysis was performed by one-way ANOVA, followed by Tukey’s multiple comparison test. The primer sequences used are shown in Supplementary Table S5.

### 4.10. Oxygen Burst and Callose Deposition Observation

We monitored H_2_O_2_ activation by staining the *N. benthamiana* leaves with 3,3′-diaminobenzidine, following a previously described method [53]. Callose deposition was detected at 24 h after agro-infiltration for *N. benthamiana*, according to Situ et al.’s methodology [42]. All experiments were repeated at least three times.

### 4.11. Genetic Transformation of Rice

The rice cultivar Nipponbare was used for the genetic transformation. The ThSCSP_12 fragment was inserted into the pBWA(V)HS plasmid, which harbors a cauliflower mosaic virus (CaMV) 35S promoter, and this constructed vector was then introduced into *A. tumefaciens* GV3101. Rice transformation was performed following a previously described method [54]. The two independent transgenic lines, ThSCSP_12OE2 and ThSCSP_12OE4, were used for the RNA-seq analysis.

### 4.12. RNA-Seq and Data Analyses

The stable T_1_ progenies of two ThSCSP_12OE lines, namely ThSCSP_12OE2 and ThSCSP_12OE4, were used for the RNA-seq analysis. The leaves of ThSCSP_12OE2 and ThSCSP_12OE4 lines were harvested at the three-leaf stage of rice plants. The leaves of WT rice line served as the control group. The cDNA library construction and Illumina sequencing (HiSeq TM 2500, San Diego, CA, USA) of RNA samples were carried out externally by the Beijing Novogene Biological Technology Co., Ltd (Beijing, China). The Illumina Hiseq platform was used for sequencing, with 125 bp paired-end sequences generated. From these raw data, we first removed any sequences that had low-quality scores or contained any adaptor sequences and stretches of -Ns. Bowtie v2.2.3 software was used to build an index of the reference genome, and paired-end sequences were aligned to the Nipponbare reference genome via TopHat v2.0.12 [55,56,57]. The number of sequences mapped to each gene was counted using HTSeq v0.6.1 [58], and the number of fragments per kilobase of transcript sequence per million (FPKM) of each gene was calculated according to the length of the gene and the number of sequence counts mapped to that gene. The DEGSeq R package was used for the analysis of DEGs (criteria: *q* < 0.05 and |log2 (fold-change)| > 1) [59]. All corresponding *p*-values were first adjusted using the method of Benjamini and Hochberg [60]. The enrichment analysis of DEGs within the KEGG pathways was performed using KOBAS (v2.0) software [61,62]. The ‘phyper’ function in R was used for the hypergeometric testing of the enrichment data, and those KEGG terms with a *p*-value < 0.05 were designated as being significantly enriched in one or more DEGs.

### 4.13. Quantitative Real-Time Reverse Transcription-Polymerase Chain Reaction

The Fungal RNA Kit (Omega, Norcross, GA, USA) and Spin Column Plant Total RNA Purification Kit (Sangon Biotech, Shanghai, China) were used to extract total RNA from *T. horrida* strains and rice or *N. benthamiana* leaves, respectively, following the instructions of each kit. The fungal conserved gene *UBQ* was used as an internal control for data normalization; for rice and *N. benthamiana* leaves, the *Actin* gene was used as an internal reference gene to determine the values for the relative expression levels. The cDNA was generated with the Transcriptor First Strand cDNA Synthesis Kit (Roche). The qRT-PCR was conducted on the Bio-Rad CFX96 Real-Time PCR System (Bio-Rad, Foster City, CA, USA) according to the manufacturer’s instructions. We used the 2^–ΔΔCt^ algorithm to calculate the relative expression levels of target genes [63]. Four biological replicates were used. Statistical analysis was performed by one-way ANOVA, followed by Tukey’s multiple comparison test. The primer sequences used are listed in Supplementary Table S5.

## 5. Conclusions

In the study, we found a secreted protein ThSCSP_12 in the biotrophic fungus *T. horrida*, likely an effector, and went on to show that it induces cell death and defense responses in the model plant *N. benthamiana*. The RAR1 protein is involved in the immune response activated by ThSCSP_12, and SP is essential for the proper functioning of ThSCSP_12. Furthermore, the expressions of multiple defense-related genes and pathways were suppressed by ThSCSP_12 in the host plant. Altogether, these results illustrate that ThSCSP_12 plays a critical role in the interactions of rice and *T. horrida* through suppression of host immunity functions. Yet, the precise molecular mechanisms through which ThSCSP_12 interacts with its target gene in host species require further study.

## Data Availability

All datasets generated for this study are included in the article/Supplementary Material. The *O. sativa* transcriptome datasets analyzed during the current study are available in the National Center for Biotechnology Information (https://www.ncbi.nlm.nih.gov (accessed on 20 April 2022)) under the accession number: PRJNA900515.

## Supplementary Materials

The following supporting information can be downloaded at: https://www.mdpi.com/article/10.3390/ijms232314752/s1.
