# Supplementary material for: ThSCSP_12: Novel Effector in Tilletia horrida That Induces Cell Death and Defense Responses in Non-Host Plants
Source: Int J Mol Sci. 2022 Nov 25;23(23):14752. doi: 10.3390/ijms232314752 (PMC9736266; doi:10.3390/ijms232314752)
Supplement: Supplementary file 1 [file ijms-23-14752-s001.zip › Supplementary Figures S1 and S2.pdf]

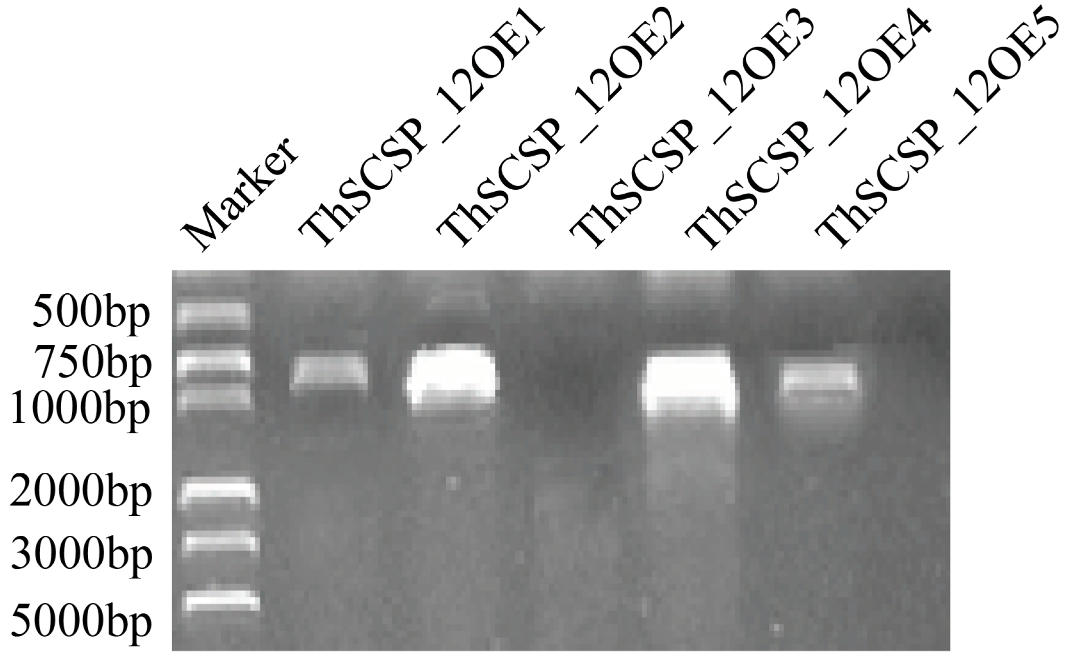

Supplementary Figure S1 Verification of the ThSCSP_12 transgenic lines by RT–PCR.

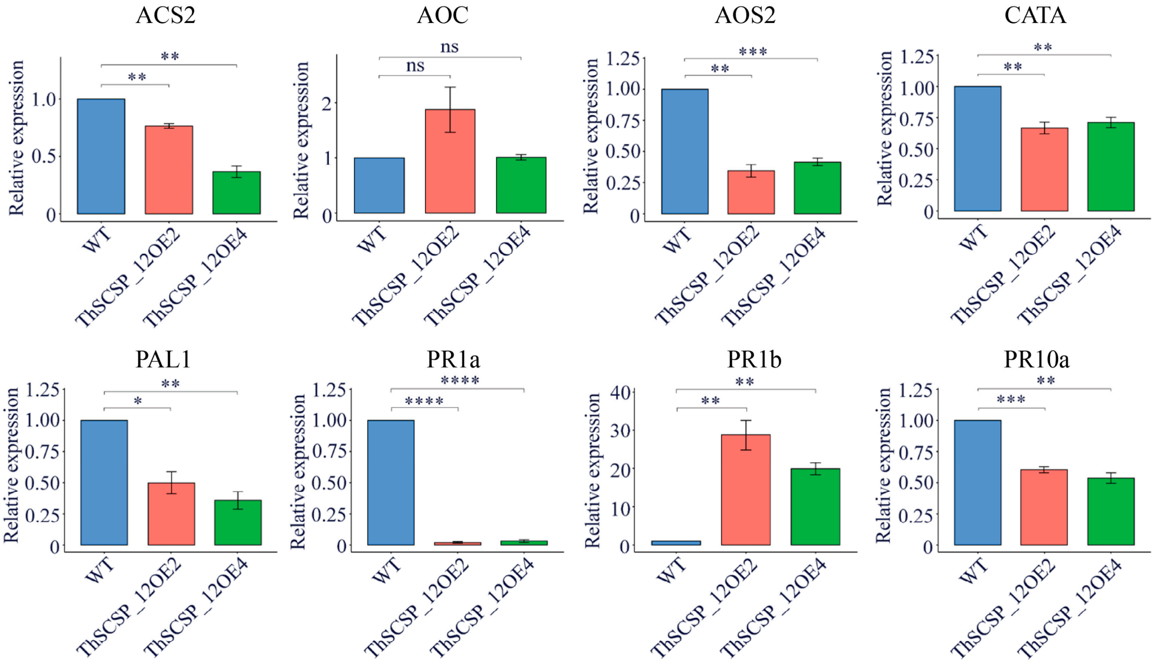

Supplementary Figure S2 Expression analysis of seven PR-related genes in the ThSCSP_12-OE2 and ThSCSP_12-OE4 plants by qRT–PCR. Statistical analysis was performed by one-way ANOVA, followed by Tukey's multiple comparison test. Error bars are the standard deviation (SD) of four independent replicates (* P < 0.05; ** P < 0.01; *** P < 0.001; **** P < 0.0001; 'ns' denotes non-significant differences between the means).
